# *Tfap2b* acts in GABAergic neurons to control sleep in mice

**DOI:** 10.1038/s41598-023-34772-x

**Published:** 2023-05-17

**Authors:** Yang Hu, Henrik Bringmann

**Affiliations:** 1 Max Planck Research Group “Sleep and Waking”, Max Planck Institute for Biophysical Chemistry, 37077 Göttingen, Germany; 2 Cellular Circuits and Systems, Biotechnology Center (BIOTEC), Center for Molecular and Cellular Bioengineering (CMCB), Technische Universität Dresden, 01307 Dresden, Germany

**Keywords:** Neuroscience, Physiology

## Abstract

Sleep is a universal state of behavioral quiescence in both vertebrates and invertebrates that is controlled by conserved genes. We previously found that AP2 transcription factors control sleep in *C. elegans, Drosophila,* and mice. Heterozygous deletion of *Tfap2b*, one of the mammalian AP2 paralogs, reduces sleep in mice. The cell types and mechanisms through which *Tfap2b* controls sleep in mammals are, however, not known. In mice, *Tfap2b* acts during early embryonic stages. In this study, we used RNA-seq to measure the gene expression changes in brains of *Tfap2b*^−/−^ embryos. Our results indicated that genes related to brain development and patterning were differentially regulated. As many sleep-promoting neurons are known to be GABAergic, we measured the expression of GAD1, GAD2 and Vgat genes in different brain areas of adult *Tfap2b*^+/−^ mice using qPCR. These experiments suggested that GABAergic genes are downregulated in the cortex, brainstem and cerebellum areas, but upregulated in the striatum. To investigate whether *Tfap2b* controls sleep through GABAergic neurons, we specifically deleted *Tfap2b* in GABAergic neurons. We recorded the EEG and EMG before and after a 6-h period of sleep deprivation and extracted the time spent in NREM and in REM sleep as well as delta and theta power to assess NREM and REM sleep, respectively. During baseline conditions, *Vgat-tfap2b*^−/−^ mice exhibited both shortened NREM and REM sleep time and reduced delta and theta power. Consistently, weaker delta and theta power were observed during rebound sleep in the *Vgat-tfap2b*^−/−^ mice after sleep deprivation. Taken together, the results indicate that *Tfap2b* in GABAergic neurons is required for normal sleep.

## Introduction

Sleep is a ubiquitous quiescence behavior that is found across species^1–3^. It can be identified using the behavioral criteria that were established by Campbell and Tobler. These criteria include the absence of voluntary movement, an increased arousal threshold, a relaxed body posture, reversibility, and homeostatic regulation^4^. In invertebrates such as *C. elegans* and *Drosophila*, sleep is characterized as a mostly quiescent behavior with reduced overall neuronal activity^3,5^. As nervous systems evolved, sleep became more complex and diversified into distinct stages^1^. In mammals, two main states of sleep have been identified, rapid eye movement sleep (REMS) and non-REM sleep (NREMS)^2^.

Functions of genes and their paralogs or orthologs in sleep behavior have been examined comparatively across invertebrates and vertebrates^6^. Such studies revealed an evolutionarily conserved role of several genes in sleep control. For example, a mutation in *Dec2* (*hDec2-P385*) was identified in humans that have a short sleep phenotype. When the equivalent mutation was introduced into transgenic *Drosophila* or mouse models, it caused a similar short sleep phenotype^7^. As a second example, a gain-of-function mutation in *SIK-3* causes an increased in sleep time in mice. Consistent with this result, sleep is reduced in *C. elegans* and *Drosophila* that carry loss-of-function mutations in *SIK-3* orthologs^8^.

As a third example of conserved sleep genes, our previous results showed that the knockout of the AP-2 transcription factor gene *aptf-1* leads to sleeplessness in *C. elegans* and knockdown of *Tfap-2* by RNAi in *Drosophila* eliminated night sleep^9,10^. There are five paralogous AP-2 proteins in mammals, called AP-2α to ε and encoded by *Tfap2a* to *e*. The structure of AP-2 transcription factor family members is evolutionarily conserved in invertebrates and vertebrates^11^. All AP-2 proteins share a highly conserved helix-span-helix domain, which mediates dimerization and DNA binding. This domain is preceded by a transactivation domain, which is more variable among the different orthologs or paralogs^11^. Heterozygous loss of function of *Tfap2b* in humans causes CHAR syndrome (CHAR, OMIM#169,100), which is characterized by abnormalities in anterior body patterning and development including patent ductus arteriosus (a congenital heart disorder), facial dysmorphism and clinodactyly (shortened or absent middle segment of the 5th finger)^12,13^. Members of two families who carry heterozygous mutations in *Tfap2b* that abrogate the DNA dimerization domain or cause mRNA degradation, had self-reported sleep disorders including sleep walking and shortened sleep during the night^12^. Previous studies by us and others have confirmed a conserved role of AP2 in sleep control^14,15^. As homozygous deletion of *Tfap2b* or *Tfap2b* is lethal in mice, we used heterozygous deletion, thus mimicking the conditions that lead to CHAR syndrome in humans. Heterozygous deletion of *Tfap2a* surprisingly caused increased sleep^14^, whereas heterozygous deletion of *Tfap2b* reduced sleep time and delta power during NREM sleep, which in combination indicate decreased sleep intensity in mammals^16,17^. REM sleep was also affected in this model as indicated by a decrease in REM sleep duration and theta power, suggesting possible implications in memory consolidation^18^. Hence*,* like *AP-2* transcriptions factors in other species, *Tfap2b* is required for normal sleep^14,15^.

Neuronal circuits and molecular mechanisms underlying sleep have been studied in both invertebrate and vertebrate animal models^3^. Our previous analysis of *aptf-1* in *C. elegans* showed that sleep can be induced by activating a single GABAergic interneuron, called RIS^9^. We showed that *aptf-1* is expressed in RIS, that its expression is highest during embryonic development, and that it is required for the functionality of this sleep-active neuron. For example, *aptf-1* is necessary for the expression of sleep-inducing FLP-11 neuropeptides, yet it was not found to be required for the expression of GABAergic genes in the sleep-active RIS neuron^19^. While FLP-11 is the most important transmitter for sleep induction in *C. elegans*^19^, it might perhaps act in concert with GABA for quiescence induction^20^. Hence, *aptf-1* acts in the GABAergic RIS neuron for this neuron to be capable of inducing sleep. In *Drosophila* and by using cells-specific RNAi, we showed that *TfAP-2* acts in neurons and the sleep loss phenotype is substantially stronger when *TfAP-2* is knocked down during development versus a knockdown that starts only in the brain at the adult stage^10^. Hence, similar to *C. elegans*, *TfAP-2* appears to have a role during development in neurons. The specific neuron type that requires this transcription factor for sleep induction and the mechanism by which it acts are, however, not known in *Drosophila*. In mice the heterozygous knockout of *Tfap2b* allowed for the identification of this gene in the control of mouse sleep^14,15^, but the cell types that require *Tfap2b* for its sleep-promoting effect and the molecular mechanisms through which *Tfap2b* affects sleep have not yet been identified.

In the larger and more complex brains of mammals, sleep manifests itself in brain oscillations initiated by neuronal networks in sleep centers like the ventrolateral preoptic area (VLPO)^21^ and the parafacial zone (PZ)^22^. These areas contain neurons that activate preferentially during sleep^23–25^. GABA has been identified as a key neural transmitter of sleep-active neurons in sleep-promoting areas. For example, GABAergic neurons of the VLPO project to arousal centers and inhibit them, thus promoting sleep^21,24,26^. Similarly, knockdown of VGAT in the mouse PZ has induced a sustaining decrease in total NREM sleep amount of around 33%^27^.

During mouse embryonic development, *Tfap2b* is broadly expressed in multiple brain areas including in the midbrain and cerebellum [Allen Developing Mouse Brain Atlas, Gene paint^28^]. In the developing cerebellum, *Tfap2b* expression is enriched in GABAergic interneurons and *Tfap2b* is necessary for specification of GABAergic function in these neurons^29^. As GABAergic neurons are key to sleep control, it seems plausible to hypothesize that *Tfap2b* is required in GABAergic neurons for normal sleep.

In this study, we wanted to identify genes that are controlled by *Tfap2b*, and to test the hypothesis that *Tfap2b* is required in GABAergic neurons for sleep. For this study, we first analyzed the effects of *Tfap2b* deletion on gene expression in mouse embryos. We also tested whether heterozygous deletion of *Tfap2b* impaired GABAergic expression in the adult mouse brain. We then generated a conditional allele and studied how homozygous *Tfap2b* deletion in GABAergic neurons affects sleep. We found that, in female mice, loss of *Tfap2b* in GABAergic neurons reduced sleep and NREMS delta power, signifying impaired sleep intensity^16^. Theta power during REMS was also decreased, indicating further sleep impairment in this mouse model. In male mice, we observed a decrease in the time spent in NREMS. These findings in combination ultimately suggest a decrease in sleep quantity and intensity in relation to *Tfap2b* loss^30,31^. Thus, *Tfap2b* in GABAergic neurons is required for sleep.

## Results

### *Tfap2b* controls the expression of genes related to embryonic brain development

**Figure 1:**
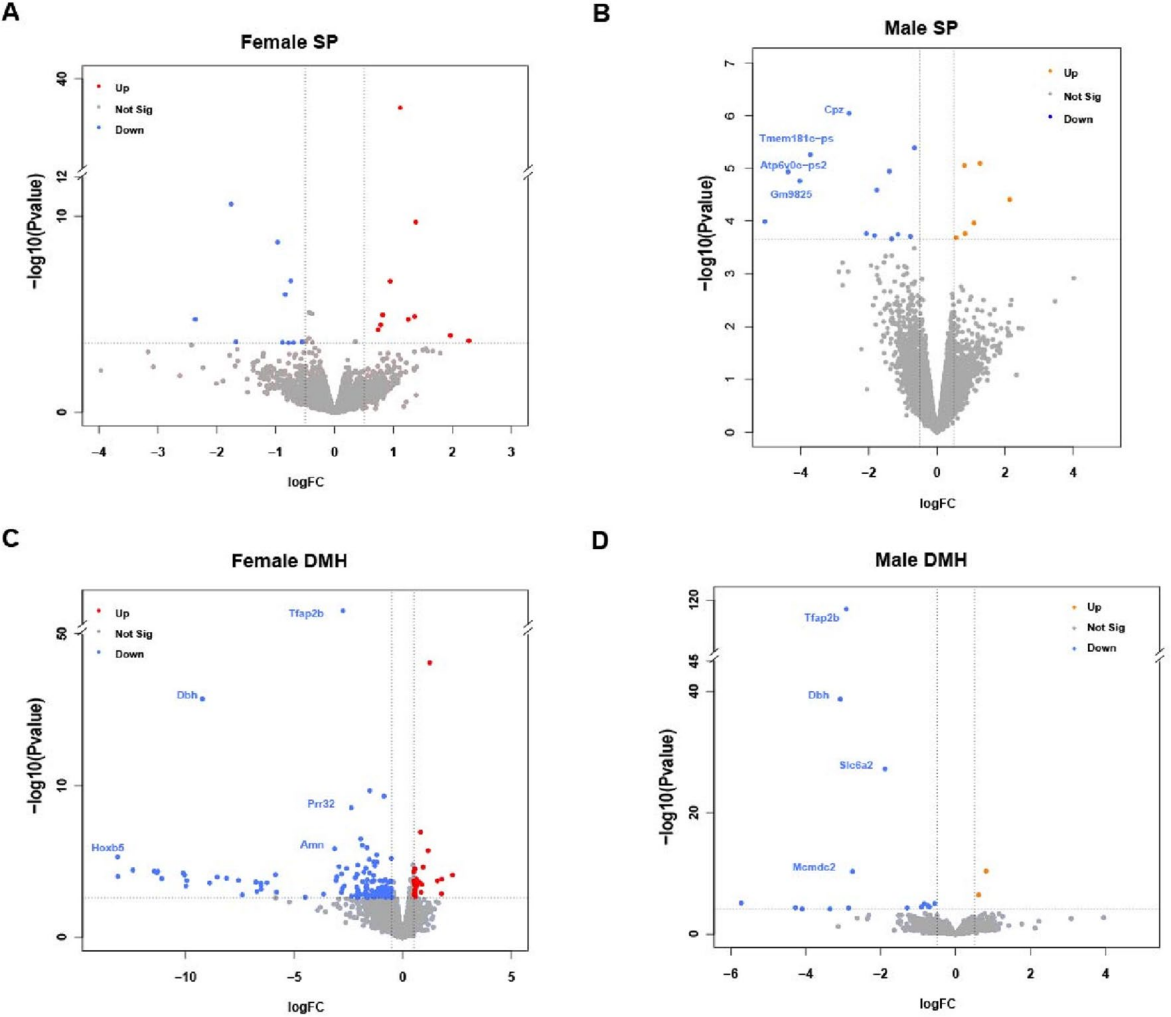
Genes differentially expressed in *Tfap2b*^−/−^ developing mouse brains. We compared gene expression between homozygous mutant embryos (E14.5) and their wild-type littermates. Volcano plots showing the significantly up-regulated (red) and down-regulated genes (blue) in female SP (**A**) and DMH (**B**). Up regulated genes are presented in orange for male (**C**) and DMH (**D**). We compared gene expression between homozygous mutant embryos (E14.5) and their wild-type littermates. Significantly changed genes as defined by an FDR (False Discovery Rate) < 0.2 and a logFC > 0.5 were highlighted and the most robustly regulated genes were labeled (FDR < 0.01; logFC > 2). Female E14.5 embryos *tfap2b*^+*/*+^, n = 3; female E14.5 embryos *tfap2b*^−/−^, n = 2 (based on PCA analysis shown in Fig. S1, the sample named “mut3” was excluded from the DE genes analysis); male E14.5 embryos *tfap2b*^+*/*+^, n = 3; male E14.5 embryos *tfap2b*, n = 3; SP, secondary prosencephalon; DMH, diencephalon, midbrain and hindbrain.

**Figure 2:**
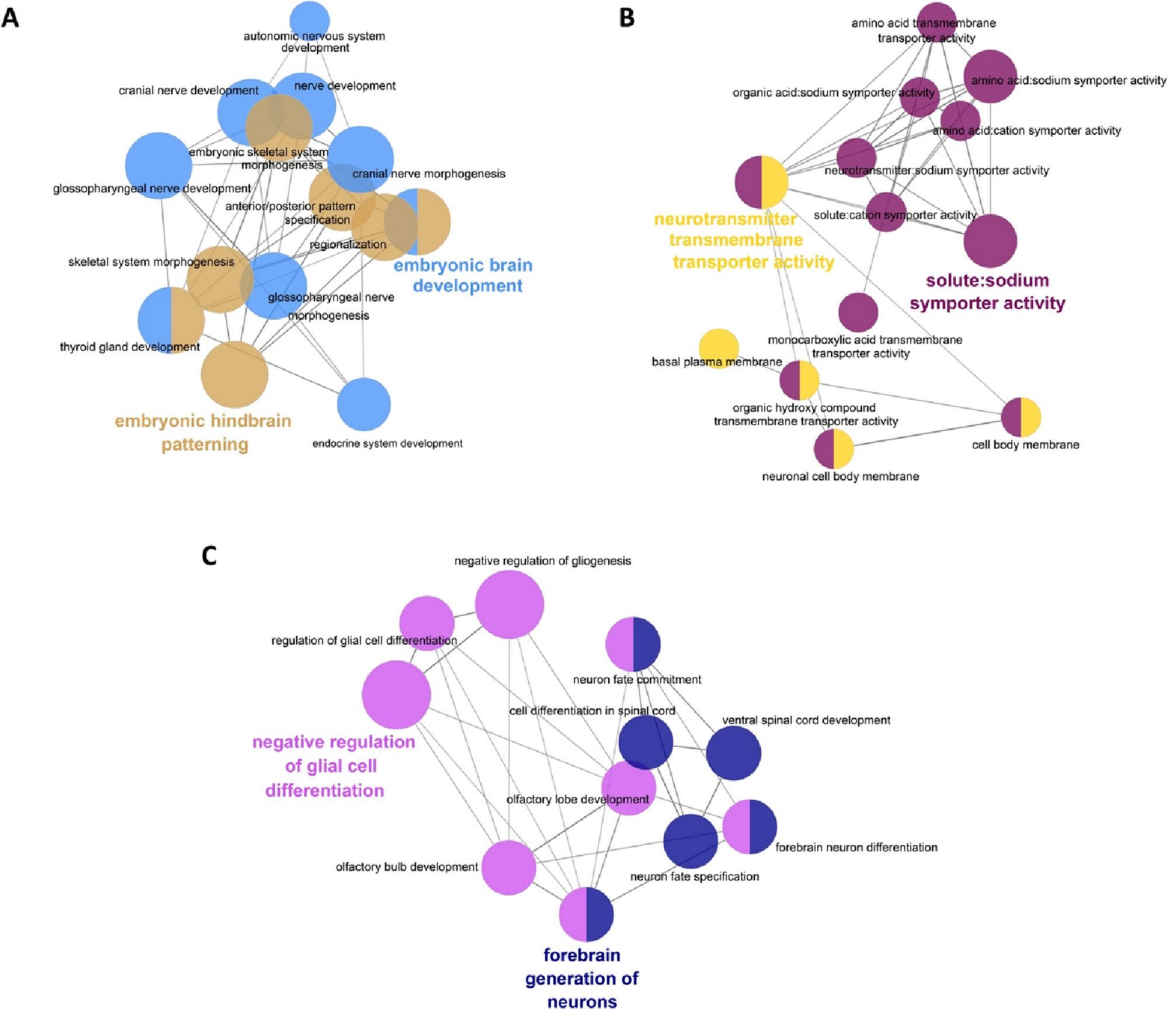
Functionally grouped gene ontology and pathway annotation networks for the genes that were differentially expressed in the *Tfap2b*^−/−^ female DMH. We classified downregulated genes by ClueGO into networks of embryonic brain development (**A**) and neurotransmission (**B**). We classified upregulated genes into major groups that are called “negative regulation of glial cell differentiation” and “forebrain generation of neurons” (**C**). We categorized all genes by biological function with GO terms as nodes where the most significant terms were highlighted. Poorly grouped terms (< 10 nodes) are not shown. Table 1 shows all of the genes found under each GO term, which are represented by different colors as specified in Table 1.

*Tfap2b* is expressed in early embryo stages, starting from E8, and is enriched in midbrain and hindbrain areas^32,33^. Complete deletion of *Tfap2b* is lethal at perinatal stages around P1-P2^34^. Here, we investigated how *Tfap2b* regulates gene expression during embryonic development. We collected *Tfap2b*^−/−^ embryos at stage E14.5 for RNA-sequencing. For this experiment, we dissected the secondary prosencephalon (SP) and diencephalon/midbrain/hindbrain (DMH) areas of female and male E14.5 embryos and compared homozygote deletion mutants with wild-type littermate controls. Overall, there were fewer differentially expressed (DE) genes in SP (Fig. 1A,B) than in DMH samples (Fig. 1C,D), and more genes were downregulated in DMH than upregulated (Fig. 1C,D). Among the highlighted genes, *Tfap2b* was significantly down regulated in both female and male DMH but not in SP (Fig. 1). The highest number of DE genes (133 genes) was found in female DMH. These genes were hence further analyzed for functionally grouped gene ontology and pathway annotation networks using ClueGO^35^ (Fig. 2). The downregulated genes were classified into two major networks, related to embryonic development and neural activity, respectively (Fig. 2A,B). All of the DE genes that are related to embryonic brain development belong to the *Hox* gene family except for *Pax8*, which is from the *Pax* family that can be recruited by AP-2 paralogs during target gene activation^36^. The enriched group of DE genes related to neurotransmission belong mostly to the solute carrier (*Slc*) family, except for *Aqp1*. Upregulated genes (31 genes) were categorized into one network consisting of neuronal generation and glial cell differentiation (Fig. 2C).

Table 1 shows all of the genes found under each GO term for the functionally grouped gene ontology and pathway annotation networks for the genes that were differentially expressed in the *Tfap2b*^−/−^ female DMH. The figure number refers to the panels where the corresponding network can be found.

**Table 1:** Differentially expressed genes from GO enrichment analysis.

GO Term	Associated Genes Found	Figure Number
Embryonic brain development (shown in light blue in Figure 2A)	*Hoxa3*, *Hoxa4*, *Hoxa5*, *Hoxa6*, *Hoxb2*, *Hoxb3*, *Hoxb4*, *Hoxb5*, *Hoxb6*, *Hoxb7*, *Hoxb8*, *Hoxc4*, *Hoxd3*, *Pax8*	Figure 2A
Embryonic hindbrain patterning (shown in brown in Figure 2A)	*Hoxa3*, *Hoxa4*, *Hoxa5*, *Hoxa6*, *Hoxb2*, *Hoxb3*, *Hoxb4*, *Hoxb5*, *Hoxb6*, *Hoxb7*, *Hoxb8*, *Hoxc4*, *Hoxc5*, *Hoxc6*, *Hoxd3*	Figure 2A
Neurotransmitter transmembrane transporter activity (shown in yellow in Figure 2B)	*Aqp1*, *Slc18a3*, *Slc6a11*, *Slc6a15*, *Slc6a2*	Figure 2B
Solute:sodium symporter activity (shown in violet in Figure 2B)	*Slc4a10*, *Slc4a5*, *Slc6a11*, *Slc6a15*, *Slc6a2*	Figure 2B
Forebrain generation of neurons (shown in dark blue in Figure 2C)	*Dlx2*, *Dlx5*, *Nr2e1*, *Sox1*	Figure 2C
Negative regulation of glial cell differentiation (shown in pink in Figure 2C)	*Dlx2*, *Hmga2*, *Nr2e1*, *Tmem98*	Figure 2C

### GABA-related gene expression is impaired in the brains of adult ***Tfap2b***^+/−^ mice

**Figure 3:**
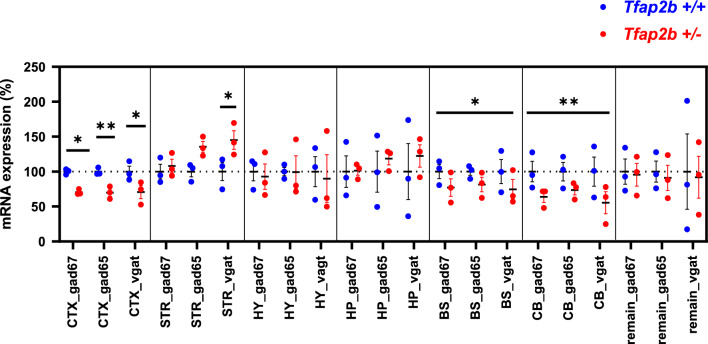
Expression of *GAD67*, *GAD65* and *Vgat* genes was altered in the brains of adult *Tfap2b*^+/−^ mice. Data were analyzed by two-way ANOVA followed by Sidak’s multiple comparisons test and were shown as the mean ± SEM, *p < 0.05, **p < 0.01. *Tfap2b*^+/+^ (n = 3) and *Tfap2b*^+/−^ (n = 3). CTX, cortex; HP, hippocampus; HY, hypothalamus; STR, striatum; BS, brainstem; CB, cerebellum. Asterisks (*) with short lines identify significance for pairwise comparisons for specific GABA-related genes in CTX and STR. In the BS and CB no significance could be found individual GABA-related genes. In the BS and CB, the asterisks with long lines identify significance in total variations of all three GABA-related genes combined.

The analysis of the role of *Tfab2b* deletion in embryos suggested a role of this transcription factor in controlling brain development. GABA is a key neurotransmitter that is required for sleep initiation and regulation, especially for the control of NREMS^22,37^, but the transcriptome analysis could not directly detect a role for *Tfap2b* in GABAergic function at the early embryonic stage. We thus asked whether *Tfab2b* might be required for the formation of GABAergic circuits that control sleep at stages that follow after the embryonic development. We hence investigated whether *Tfap2b* regulates GABA-related gene expression in the brain of adult mice. As homozygous deletion of *Tfab2b* is perinatally lethal, we used *Tfap2b*^+/−^ mice and compared their GABA gene expression to their wild-type littermate controls. We dissected the brain cortex (CTX); the hippocampus (HP); the hypothalamus (HY); the striatum (STR); the brainstem (BS); and the cerebellum (CB), and isolated separately the RNA of each of these parts. In each of these RNA samples we quantified the expression of the glutamate decarboxylase genes *GAD67* and *GAD65,* and the GABA transporter gene *Vgat* using qPCR. We observed altered expression changes in several brain areas of *Tfap2b*^+/−^ mice compared to their controls (Fig. 3). Specifically, there was an overall down regulation of all three genes in CTX, BS and CB areas. Interestingly, an up-regulation of *Vgat* was observed in the STR. These results suggest that *Tfap2b* plays a role regulating the expression of GABA-related genes.

### Shortened sleep and reduced sleep intensity in female ***Vgat-tfap2b***^−/−^ mice

Our previous results showed that heterozygous deletion of *Tfap2b* in all cells of the mice shortened sleep length and reduced NREM delta and REMS theta power, indicating lower NREM sleep intensity and impaired REMS^14^. Our qPCR results above (Fig. 3) showed that the expression of GABAergic genes is impaired in the *Tfap2b*^+/−^ mouse brain. GABAergic neurons are known to have sleep-promoting functions. For example, ablating GABAergic neurons in the parafacial zone (PZ) or ventrolateral preoptic area (VLPO) can reduce NREMS^22,38,39^. To investigate whether *Tfap2b* acts in GABAergic neurons for sleep control, we deleted *Tfap2b* specifically in GABAergic neurons. For this experiment, we generated a conditional allele of *Tfap2b*, *Tfap2b*^*tm1c*^ (see methods^40^), and crossed it with a Cre recombinase driven by the *Vgat* promoter^41^. To quantify sleep in these mice, we used electroencephalogram (EEG) analysis (see methods) and measured sleep over the 24-h Zeitgeber Time (ZT) course for 48 h. We compared the conditional knockdown animals with littermates that carried either the Cre recombinase or that were homozygous for the floxed allele.

**Figure 4:**
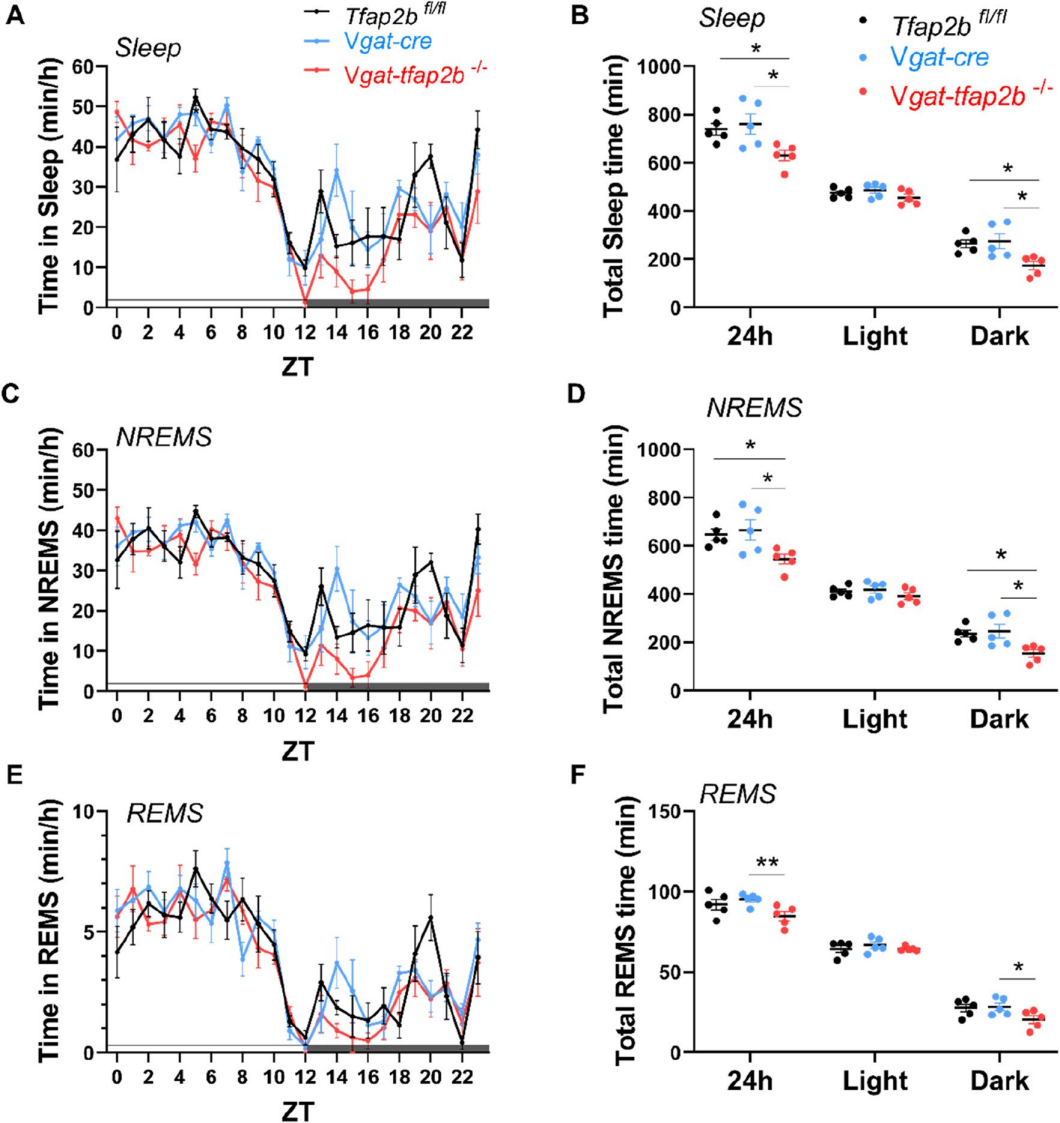
Sleep time is shortened in female GABA neuron-specific *Tfap2b* homozygous knockdowns. (**A**) Time spent in sleep in female *Tfap2b*^*fl/fl*^, *Vgat-cre*, *Vgat-tfap2b*^−/−^ mice over the Zeitgeber time (ZT) course. (**B**) Sleep quantity during 24 h, light and dark phase. ZT course NREMS quantification (**C**) and total NREMS amount during 24 h, light and dark phase (**D**). REMS quantification over the ZT course (**E**) and during 24 h, light and dark phases (**F**). All data were analyzed by two-way ANOVA followed by Sidak’s multiple comparisons test and were shown as the mean ± SEM. Significant pairwise comparisons were marked with *P < 0.05, **P < 0.01. Female *Tfap2b*^*fl/fl*^ (n = 5), *Vgat-cre* (n = 5), *Vgat-tfap2b*^−/−^ (n = 5).

**Figure 5:**
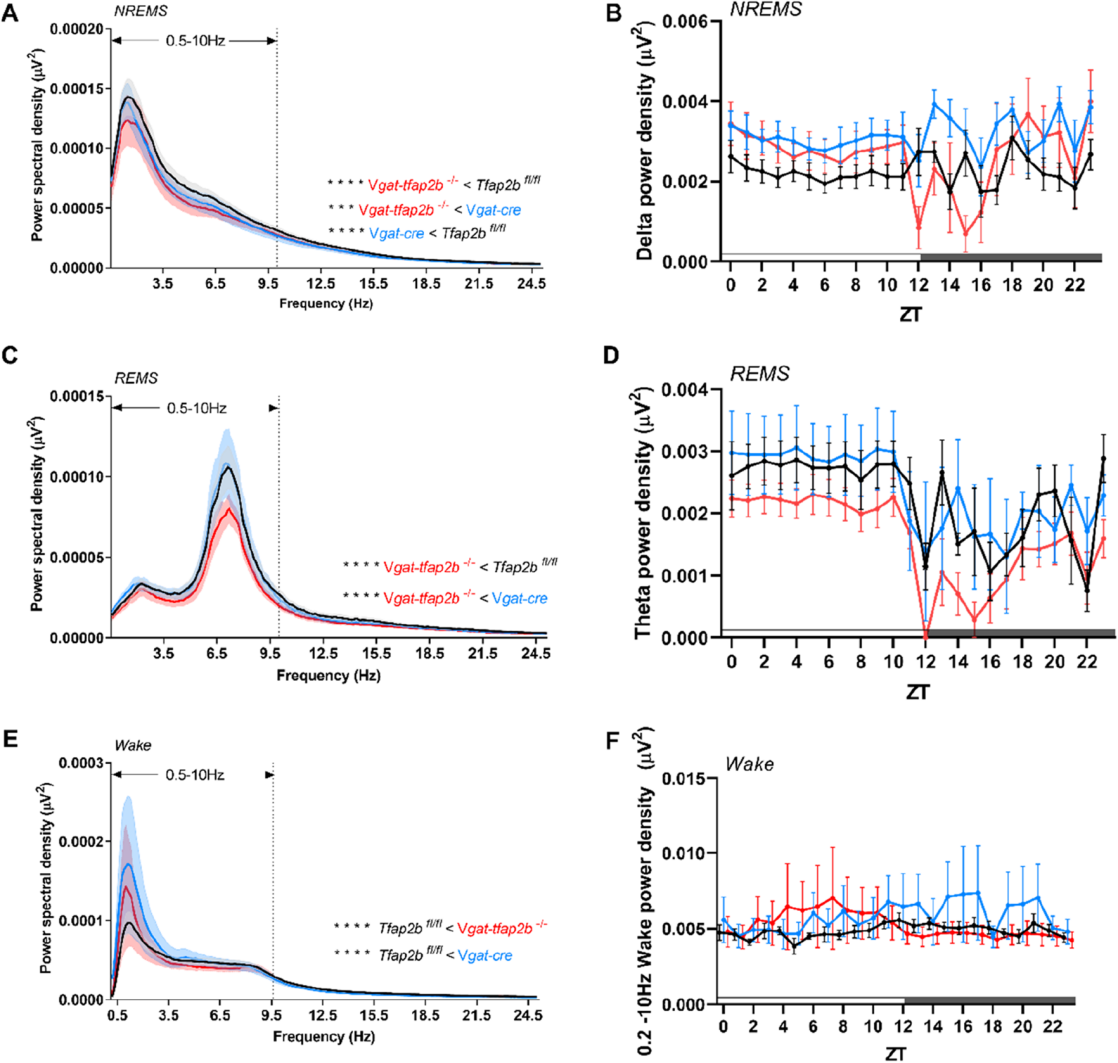
Reduced delta and theta power during NREM and REM sleep, respectively, in female *Vgat-tfap2b*^−/−^ mice. NREMS power spectra in 24 h scale (**A**) and delta power (0.5 – 4.0 Hz) changes over ZT course (**B**). REMS power spectra in 24 h scale (**C**) and theta power (5 – 10 Hz) changes over ZT course (**D**). Wake power spectra and 0.2—10 Hz power changes over ZT course. Data in (**A**, **C**, **E**) were analyzed using Friedman test followed by Dunn’s multiple comparisons test. Data in (**B**, **D**, **F**) were analyzed by two-way ANOVA followed by Sidak’s multiple comparisons test and were shown as the mean ± SEM. Highlighted is the 0.5–10 Hz frequency band (**A**, **C**, **E**), which was used for statistical analysis. Significant pairwise comparisons of *Tfap2b*^*fl/fl*^ vs. *Vgat-tfap2b*^−/−^ were marked with ***P < 0.001, ****P < 0.0001. Female *Tfap2b*^*fl/fl*^ (n = 5), *Vgat-cre* (n = 5), *Vgat-tfap2b*^−/−^ (n = 5).

The EEG analysis of female *Vgat-tfap2b*^−/−^ mice revealed that total sleep time was decreased by approximately 120 min per 24-h day (Fig. 4A,B). NREMS and REMS time decreased most strongly within the first half of the dark phase (Fig. 4C–F). In addition to the shortened sleep time, both delta and theta power were reduced in the female *Vgat-tfap2b*^−/−^ mice (Fig. 5A–D). Plotting power versus time of the day suggested that delta and theta power were most strongly reduced during the first half of the dark phase (Fig. 5B,D). Hence, both sleep time as well as delta and theta power are mostly reduced during the first half of the dark phase. A small difference between the two control groups was observed in the spectrum power analysis of the wake state (Fig. 5E,F). In the *Tfap2b*^fl/fl^ control the EEG spectral power at slower frequency was lower than that in the *Vgat-Cre* control. Wake spectral power intensity of *Vgat-tfap2b*^−/−^ knockouts fell in between the controls. The wake power intensity of wakefulness thus seems to change independently of the slow-wave intensity in sleep. The sleep and wake bout lengths in the mutant group were not affected by the altered sleep or wake spectral power (Fig. S2). In summary, the female *Vgat-tfap2b*^−/−^ mice displayed reduced sleep time and sleep depth.

### Reduced homeostatic sleep response in female ***Vgat-tfap2b***^−/−^ mice

Sleep pressure, or sleep need, accumulates as wake time progresses and dissipates during sleep^42^. The dynamics of sleep homeostasis is best characterized by spectral power changes during rebound sleep^43,44^. To determine the homeostatic sleep response, mice were deprived of sleep (SD) for 6 h after BSL recording and recovery sleep was measured for another 48 h. Following SD, a trend of increased sleep was observed during the first recovery day (R1) in both control and knockout groups with the main effect occurring during the dark phase (Fig. S3). The NREMS and REMS architecture was restored to baseline levels in all groups within the second day of recovery (R2) (Fig. S4). Spectral power analysis during R1 revealed a delta power increase compared to baseline sleep power within the equivalent ZT range in all groups tested. During R2, the delta power had returned to baseline levels.

**Figure 6:**
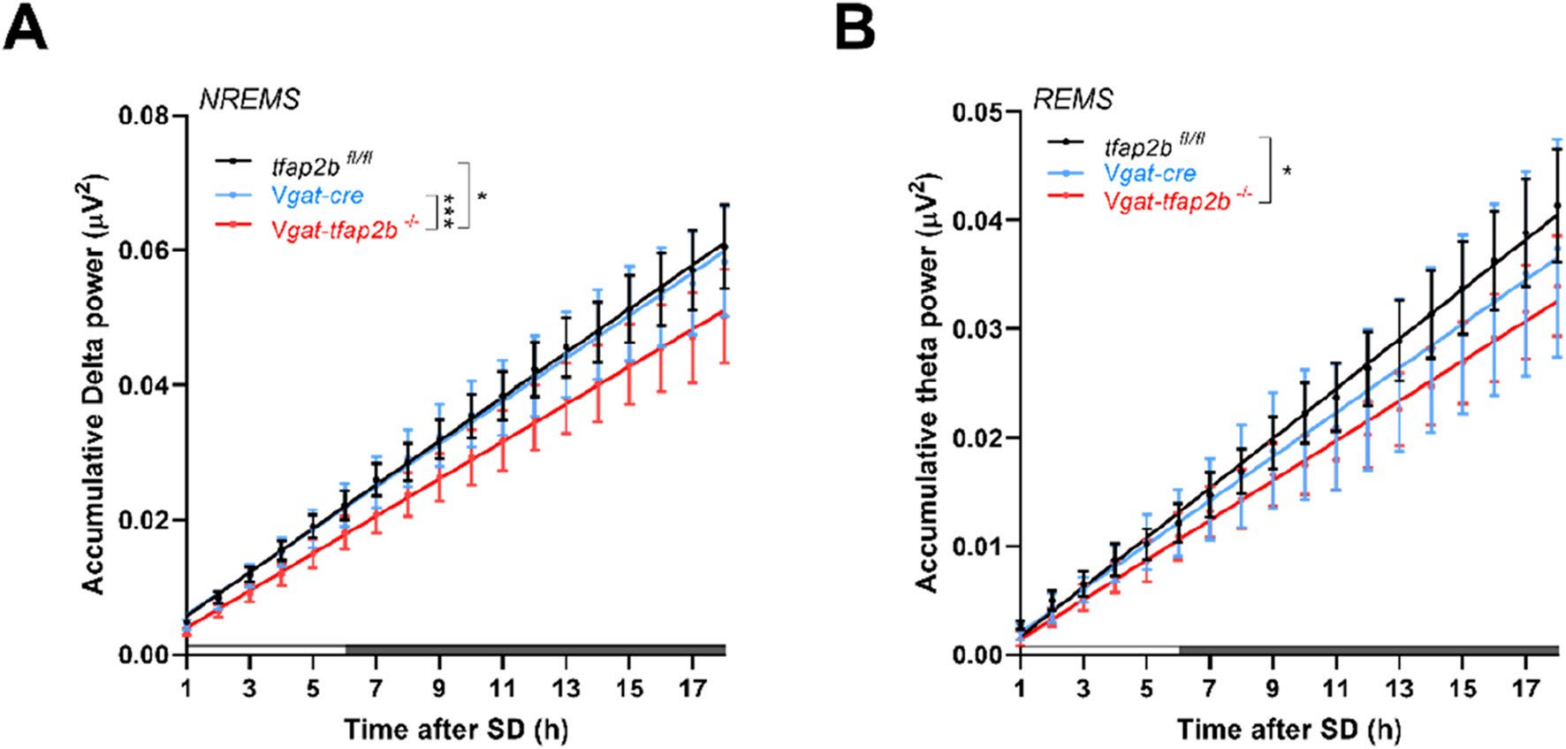
Delta and theta power during rebound sleep are weaker in female *Vgat-tfap2b*^−/−^ mice. Linear regression plots of accumulative delta (**A**) and theta power (**B**) following 6 h of SD. Equality of slopes or intercepts was tested by linear regression analysis. Significant differences were detected between groups and marked with *P < 0.05, ***P < 0.001. Female *Tfap2b*^*fl/fl*^ (n = 5), *Vgat-cre* (n = 5), *Vgat-tfap2b*^−/−^ (n = 5).

The theta power rebound during REMS showed a statistically significant increase in female *Vgat-tfap2b*^−/−^, but the changes in control groups did not reach statistical significance (Fig. S4). We therefore calculated the accumulative effect of the theta and delta power rebound for all genotypes. The cumulative delta and theta power was calculated for NREMS and REMS, respectively. Results of both delta and theta oscillations showed that the recovery sleep in female *Vgat-tfap2b*^−/−^ mice was slower and weaker than their control littermates (Fig. 6). Taken together, the homeostatic sleep response was reduced in female *Vgat-tfap2b*^−/−^ mice.

### Male ***Vgat-tfap2b***^−/−^ mice spent less time in NREM sleep

**Figure 7:**
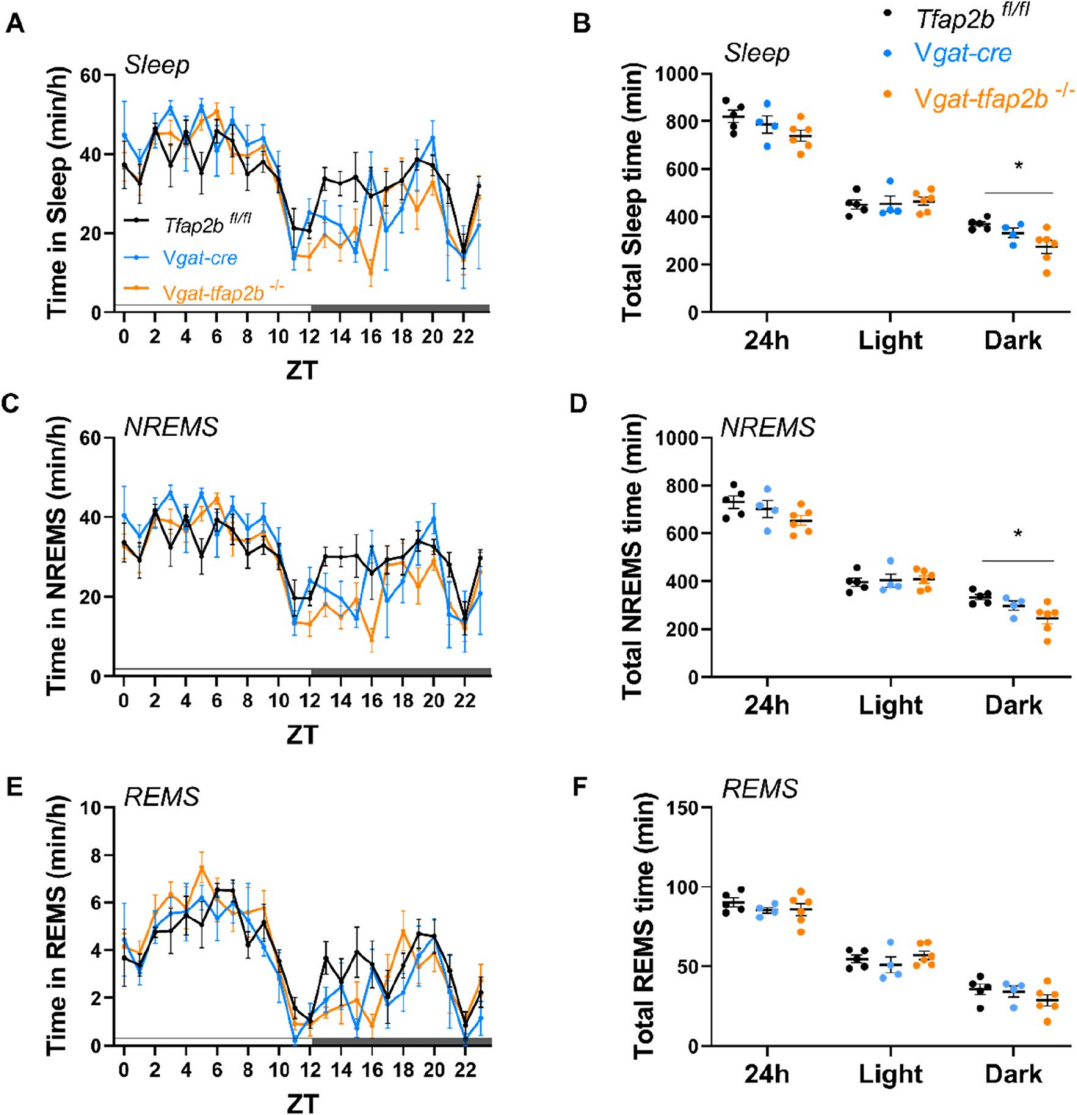
Sleep time is shortened in male GABAergic specific *Tfap2b* homozygous knockouts in baseline recording. (**A**) Sleep quantity changes in male *Tfap2b*^*fl/fl*^, *Vgat-cre*, *Vgat-tfap2b*^−/−^ mice over the Zeitgeber time (ZT) course. (**B**) Sleep quantity during 24 h, light and dark phase. (**C**) ZT course NREMS quantification and (**D**) total NREMS amount during 24 h, light and dark phase. REMS quantification over the ZT course (**E**) and during 24 h, light and dark phases (**F**). All data were analyzed by two-way ANOVA followed by Sidak’s multiple comparisons test and were shown as the mean ± SEM. Significant pairwise comparisons were marked with *P < 0.05. Male *Tfap2b*^*fl/fl*^ (n = 5), *Vgat-cre* (n = 4), *Vgat-tfap2b*^−/−^ (n = 6).

We showed here that knockdown of *Tfap2b* in GABAergic neurons reduces sleep in female mice. We also measured EEG/EMG in male mice. Consistent with our results in females, the total sleep time and NREMS were also reduced in male mutants (Fig. 7, S5). The effect of GABAergic neuron knockdown of *Tfap2b* extended to the rebound sleep that is observed following sleep deprivation, but less strongly than in females (Fig. S6). Hence *Tfap2b* in GABAergic neurons plays a role in both, baseline and rebound sleep following sleep deprivation. Thus, *Tfap2b* is required in GABAergic neurons also for sleep in males.

The delta and theta power are, however, increased in *Vgat-tfap2b*^−/−^ males (Fig. S7–S9). Male *Vgat-cre* control mice also displayed a stronger delta and theta power, suggesting that the *CRE* gene might have caused the power increase. The increased delta and theta power phenotype that is associated with the expression of CRE may have masked potential effects caused by *Tfap2b* knockdown. The effects of *Tfap2b* knockdown in GABAergic neurons on delta and theta power thus cannot be clearly interpreted in *Vgat-tfap2b*^−/−^ males. In the power analysis of the male groups, the two controls, *Vgat* and *tfap2b*^fl/fl^, behaved differently albeit a similar genetic background and experimental conditions*. Vgat-cre* mice have been previously used in combinations with stereotaxic AAV injection^45–47^. In these conditions there is one control group involved in each experiment, whereas in the case of breeding conditional knockouts there are two parental control strains that need to be investigated, which increases the likelihood that one parental strain has altered sleep. It hence appears that *CRE* recombinase expression might only increase delta and theta power in males, but not in females. To clearly test the delta and theta power changes that are induced by knockdown of *Tfap2b* on sleep power, further studies would be required that, for example, employ a GABA-specific CRE driver that does not interfere with sleep power in males.

## Discussion

AP-2β is expressed in migrating neural crest cells and the expression continues in neural tissues, facial mesenchyme and peripheral system^48^. Homozygous loss-of-function mutation in *Tfap2b* is perinatal lethal in mice, which is due to renal malfunction^34^. In our study, a significant decrease of *Tfap2b* was detected in mutant DMH but not in SP. ISH data from other studies revealed a similar pattern that *Tfap2b* expression is more robust in mid- and hindbrain areas compared with forebrain^33^. Thus, it is expected that more DEGs related to embryonic brain development identified in DMH than SP. This supports the view that *Tfap2b* plays a prominent role in neuronal development in the DMH. Consistent with our analysis, an in vivo study has demonstrated that *Tfap2b* is required for the differentiation of noradrenergic neurons^49^. In addition, another study showed that *Tfap2b* mediates the differentiation of GABAergic interneurons in the cerebellum^29^. Thus, consistently across species, *Tfap2b* homologs are required for brain development. The impaired GABAergic gene expression in multiple brain areas in our *Tfap2b*^+/−^ model suggests that this transcription factor might control GABAergic function not only in the cerebellum but in multiple brain regions.

We have mapped a sleep-promoting effect of *Tfap2b* onto GABAergic neurons. While we have mapped an effect of *Tfap2b* to GABAergic neurons, the specific populations of GABAergic neurons in which *Tfap2b* acts to promote sleep remain unidentified. GABA is widely used in the nervous system. It would thus be interesting in future studies to further map the function of *Tfap2b* onto specific subpopulations of GABAergic neurons. It would be particularly interesting to test whether *Tfap2b* is required for the development or functioning of sleep-promoting neurons. It is equally possible that *Tfap2b* is required for sleep in neurons that are not sleep active. This is particularly relevant as GABAergic neurons control many functions such as motor control^50^, thermoregulation^51,52^, pain^53^, memory and anxiety^54^. Hence, effects on sleep could potentially be indirect. Also, it is possible that *Tfap2b* acts not only in GABAergic neurons but also in other neuron types. A mapping of the sleep function of *Tfap2b* on specific populations of neurons would also facilitate the identification of the molecular function of *Tfap2b* in these specific neuronal populations.

In the mammalian brain there are both, populations of GABAergic neurons that promote sleep as well as populations of GABAergic neurons that promote wakefulness. For example, chemogenetic activation of GABAergic neurons in the basal forebrain (BF) promotes wakefulness^55,56^, whereas chemogenetic activation of populations of GABAergic neurons in the VLPO and PZ promote sleep^22,24^. Also, brain areas that control sleep and wakefulness may contain mixed populations of both sleep-promoting neurons and wake-promoting GABAergic neurons. For example, the BF and the VLPO contain such mixed populations of GABAergic neurons, some of which are wake active and others are sleep active^55,57^. Some approaches have been established to specifically target subpopulations of such GABAergic neurons. For example, a specific subpopulation of VLPO GABA neurons, which have projections to the tuberomammillary nucleus (TMN), can be targeted by retrograde labeling, which demonstrated that this specific neuronal subpopulation promotes sleep^24^. Hence, some populations of GABAergic neurons can promote sleep, whereas other populations of GABAergic neurons might function to inhibit sleep-promoting neurons^58,59^. Hence, the identification of the molecular function of *Tfap2b* in sleep will ultimately require more specific manipulations of *Tfap2b.*

We did not detect a defect in GABAergic gene expression in the hypothalamus, which contains sleep-active neurons in the VLPO^58^. As brain regions such as the hypothalamus contain many different populations of different neuron types, this does not however exclude the possibility that *Tfap2b* might still be playing a role in sleep-promoting areas within the hypothalamus and it does not exclude the possibility that *Tfap2b* controls GABA-related gene expression in subsets of neurons. Hence, the effects of *Tfap2b* knockdown in GABAergic neurons could be different for different populations of GABAergic neurons, and the effects on GABAergic gene expression could be restricted to a small subset of GABAergic neurons within the hypothalamus such as sleep-active neurons of the VLPO or other neurons and might thus be difficult to detect by analyzing gene expression at the level of the entire hypothalamus.

It remains unclear why *Tfap2b* is required for sleep in GABAergic neurons. *Tfap2b* has been shown to control the expression of GABA-related genes, but it is not known whether this function also underlies its role in sleep. In *C. elegans*, the *Tfap2b* homolog *aptf-1* does not seem to control sleep by controlling the expression of GABA-related genes, but through the control of expression of a neuropeptide gene^19^. While the idea that *Tfap2b* is required for sleep by controlling GABA-related gene expression seems plausible, it is likely that *Tfap2b* is playing a role in sleep regulation through other genes.

We have knocked down *Tfap2b* during development. It hence remains unclear whether *Tfap2b* is required specifically during development, i.e. for the normal creation of neuronal circuits that are involved in the control of sleep or whether *Tfap2b* is required for the functioning of mature neurons and circuits. Such adult-specific inhibition of *Tfap2b* could for example be tested by tamoxifen-inducible CRE^60^, by injecting CRE-expressing AAV into specific brain areas in the adult^61^, or by injecting AAV containing floxed shRNA against *Tfap2b* into specific brain regions such as the VLPO in animals expressing CRE in GABAergic neurons^62,63^. Such additional experiments will be instrumental also in identifying specific neuronal populations in which *Tfap2b* acts. Once specific populations of neurons are identified in which *Tfap2b* is required for sleep, transcriptomic and electrophysiological analysis of these specific neurons can be carried out with and without the knockdown of *Tfap2b*.

Previous studies have investigated the function of other transcription factors in sleep regulation^7,64,65^. For example, another transcription factor, *Lhx6*, exerts a mild sleep-reducing effect via GABA-releasing neurons in the zona incerta of the lateral hypothalamus^64,66^. Thus, *Tfap2b* might be one of the genetic factors that regulates sleep-initiating GABAergic neurons. Here, and consistent with our work in *C. elegans*, we find a role of *Tfap2b* in controlling sleep by acting in GABAergic neurons.

## Methods

### Authorizations to carry out experiments with live mice

The LAVES (Niedersächsisches Landesamt für Verbraucherschutz und Lebensmittelsicherheit) approved the experiments with live mice. All experiments were performed in accordance with relevant EU and German guidelines and regulations. The authors complied with the ARRIVE guidelines.

### Animals

Embryos carrying the *Tfap2b*^*tm1a*^ allele (knock-out first *tm1a* allele) were purchased from EMMA. We first crossed *Tfap2b*^*tm1a*^ with the FLP deleter strain 129S4/SvJaeSor-*Gt(ROSA)26Sor*^*tm1(FLP1)Dym*^/J (#003,946, Jackson laboratory). The FLP recombinase removed the trapping cassette from the *Tfap2b*^*tm1a*^ allele, therefore converted the *tm1a* allele to the conditional (floxed) allele *Tfap2b*^*tm1c*^ (here we call *Tfap2b*^*fl*^). The deletion was verified by PCR.

The V*gat-cre* mouse line B6J.129S6(FVB)-*Slc32a1*^*tm2(cre)Lowl*^/MwarJ(#028,862, Jackson laboratory) was a gift from Professor Nils Brose from Max Planck Institute of Experimental Medicine.

To conditionally delete *Tfap2b* from GABAergic neurons, we first crossed *tfap2b*^*fl/*+^ mice with V*gat-cre* mice to obtain V*gat*-*tfap2b*^*-/*+^ mice. V*gat*-*tfap2*^+/−^ mice were then bred to *Tfap2b*^*fl/*+^ mice to generate homozygous knockouts (V*gat*-*tfap2b*^−/−^) and the respective littermate controls. We used the *Tfap2b*^*fl/fl*^ and heterozygous V*gat-cre* mice as the control groups in this experiment. Both male and female mice were used in the EEG recording.

*Tfap2b* knockout (*Tfap2b*^+/−^) mice were provided by Markus Moser [Max Planck Institute (MPI) for Biochemistry] with PGK-neo cassette inserted into exon four of the *Tfap2b* gene^34^. On embryonic day (E) 14.5, mouse embryos were harvested from mother *Tfap2b*^+/−^ mice, which had been mated with the male mice of *Tfap2b*^+/−^. The brains were extracted and dissected into secondary prosencephalon (SP) and diencephalon/midbrain/hindbrain (DMH) areas. All tissues were snap-frozen and stored at − 80 °C before use.

### Genotyping

Ear biopsies of mice or tails from embryos were collected and genomic DNA was extracted as described previously^14^. Briefly, samples were lysed in 50 µl of PBND buffer (50 mM KCl, 10 mM Tris HCl pH 8.3, 0.1 mg/mL MgCl_2_·6H_2_O, 0.1 mg/mL Gelatin, 0.45% NP-40, 0.45% Tween20) with 2.5 µl proteinase K (#P8107S, New England BioLabs) freshly added. The samples were incubated in a thermomixer at 55 °C overnight until samples were completely lysed. The proteinase K was then deactivated in the samples by incubation at 85 °C for 45 min. The lysate was then centrifuged at 6000 × g and the supernatant was used for PCR. Primers used for genotyping and PCR conditions are listed in Table S1. Tails from all embryos were measured for determining the genotype and the sex of the individuals.

### RNA-sequencing

Brains from E14.5 embryos were extracted and dissected into prosencephalon (SP) and diencephalon/midbrain/hindbrain (DMH) areas from three female and three male individuals per sex and genotype. Thus, in total, we used 12 embryos. We isolated RNA from each sample individually according to the manufacture’s protocol (Qiagen RNeasy Mini plus). RNA sequencing was carried out by Bernd Timmermann and Stefan Börno at the sequencing facility of the Max Planck Institute for Molecular Genetics, Berlin, according to their protocol, which we have described previously in^14^ and which is cited here: After quality control using Agilent’s Bioanalyzer, sequencing libraries were prepared from 500 ng of total RNA per sample following Roche’s stranded “KAPA RNA HyperPrep” library preparation protocol for single indexed Illumina libraries: First the polyA-RNA fraction was enriched using oligo-dT-probed paramagnetic beads. Enriched RNA was heat-fragmented and subjected to first strand synthesis using random priming. The second strand was synthesized incorporating dUTP instead of dTTP to preserve strand information. After A-tailing, Illumina sequencing compatible adapters were ligated. Following bead-based clean-up steps the libraries were amplified using 11 cycles of PCR. Library quality and size was checked with qBit, Agilent Bioanalyzer and qPCR. Sequencing was carried out on an Illumina NovaSeq2 system in PE100bp mode yielding around 50 million fragments per sample. For the analysis, we compared *Tfap2b*^+*/*+^ and *Vgat-Tfap2b*^−/−^.

### Quantitative PCR

Brains from adult *Tfap2b*^+/−^ and their littermate controls (*Tfap2b*^+*/*+^) were extracted and dissected into different brain areas, which were: cortex, striatum, hypothalamus, hippocampus, brainstem, cerebellum and all of the remaining brain areas. Total RNA was extracted from each sample using TRIzol and was reverse transcribed into cDNA using the high-capacity cDNA RT kit (Applied Biosystems) according to the manufacturer’s instructions. Reverse transcription was performed at 25 °C for 10 min, 37 °C for 120 min, 85 °C for 5 min and samples were kept in 4 °C or − 20 °C until use. Subsequently, the cDNA levels were quantified using the Fast SYBR Green Master Mix (Applied Biosystems). mRNA expression was analyzed using the 2^-ΔΔCq^ method where the control was normalized to 100%, and the samples from the mutants were compared with their control. The primers used and qPCR conditions are listed in Table S2.

### Surgeries

Female and male mice were used for the EEG experiment and the surgeries were performed as described previously^14^. Briefly, mice were anesthetized using isoflurane throughout the operation. Two EEG recording electrodes (Bilaney Consultants GmbH) were implanted bilaterally over frontal cortex (AP + 1.5 mm from bregma; mediolateral ± 1.7 mm), and one recording electrode was implanted over the right parietal lobe (AP + 1.5 mm from lambda; mediolateral + 1.7 mm). Two reference electrodes were placed bilaterally over the cerebellum (AP − 1.5 mm from lambda; mediolateral ± 1.7 mm). One subcutaneous pad was inserted to the nuchal muscle to record the electromyogram (EMG). The wires attached to the electrodes and subcutaneous pad were assembled into a pedestal (PlasticsOne, Bilaney Consultants GmbH). The surgical exposure was covered with dental cement. At least 8 days were allowed for recovering before connecting to the recording cable. All mice were given 48 h to acclimate to the recording cylinder.

### EEG recording setups and data analysis

As previously described, the recording was performed in condition under 12 h/12 h (Light/Dark) cycles with light-on at 6AM (ZT0) to 6PM (ZT12) every day. EEG/EMG signals were recorded at a sampling rate of 2000 Hz (Multi Channel Systems MCS GmbH) starting from ZT0 for 48 h as baseline recording, followed by a 6 h-sleep-deprivation from ZT0 to ZT6 by gentle handling^67^ and novel objects interaction^68^, subsequently a recovery sleep of 48 h. EEG/EMG data were analyzed in a semi-automatic way that the signals were first trained by a human scorer using Sirenia Sleep Pro (Pinnacle Technologies, Lawrence, KS) at 10 s epoch and then analyzed by a MatLab-based custom-written system^69^, followed by visual inspection by the same scorer. NREMS was scored based on the presence of the slow activity (0.5–4 Hz) with high amplitude (delta waves) and quiet EMG activity. REMS was characterized as theta activity (6–10 Hz) with a slightly higher frequency than delta waves and EMG atonia. Time spent in each vigilance state was calculated and plotted over the 24 h course or as total hours during the 24 h/ light/ dark phase for each genotype. For 48 h of baseline recording, values were averaged and plotted as a 24 h scale. Spectral analysis was performed as described by Kent *et* al.^70^ Raw power data of the vigilance states were presented based on the distribution of power in 0.1 Hz windows or frequency classes. Accumulative spectral power was plotted to visualize the rebound power changes in NREMS and REMS. All power data were expressed as averaged values from frontal and temporal recordings. The scorer was blind to the genotypes of all mice during the analysis.

### Statistics

Statistics analysis and graph plotting were performed using SPSS or GraphPad Prism 8. Shapiro–Wilk Normality test was used to analyze the distribution of data. Levene’s test was adopted to test whether variances were equal. Parametric tests were performed for the datasets showed a Gaussian distribution. Specifically, for comparisons between two groups, two-tailed paired/unpaired t-test was performed. As for multi-group comparisons, ANOVA followed by Sidak’s multiple comparisons was performed. Nonparametric tests were used for the dataset that was not normally distributed or the variances were not equal. In such circumstances, Mann–Whitney test or Wilcoxon signed-rank were used for two-group comparison, or Friedman test followed by Dunn’s multiple comparisons test was performed for multi-group comparisons. Linear regression analysis was performed to plot accumulative spectral power, and equality of slopes or intercepts was calculated to examine the difference between genotypes. ClueGO^35^ was used in the network analysis of DE genes from RNA-analysis.

## Supplementary Information

Supplementary Information.

## Data Availability

The authors state that all data necessary for confirming the conclusions presented in the article are represented fully within the article. All data are available at Dryad (https://datadryad.org/stash/share/hzwhRUy0u5DTwk-CK4tcRFq1o54oruMyWXpqJaPQeUU; 10.5061/dryad.05qfttf6c). Raw RNA-seq data have been deposited in NCBI’s Gene Expression Omnibus^71^ and are available through GEO Series accession number GSE217724 (https://www.ncbi.nlm.nih.gov/geo/query/acc.cgi?acc=GSE217724).
