# Supplementary material for: Tfap2b acts in GABAergic neurons to control sleep in mice
Source: Sci Rep. 2023 May 17;13:8026. doi: 10.1038/s41598-023-34772-x (PMC10192351; doi:10.1038/s41598-023-34772-x)
Supplement: Supplementary file 1 — Supplementary Information. [file 41598_2023_34772_MOESM1_ESM.pdf]

Tfap2b acts in GABAergic neurons to control sleep in mice

Authors: Yang Hu[1], Henrik Bringmann[1,2]

Supplemental Information

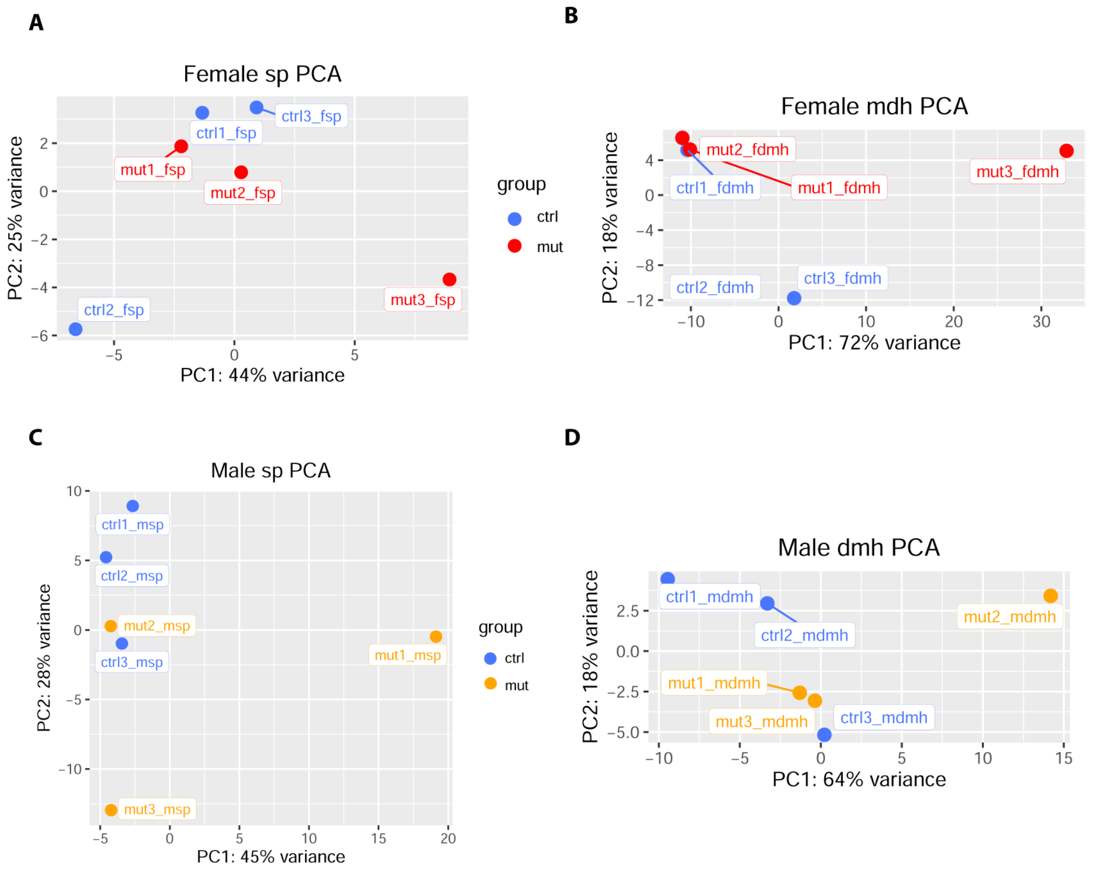

Figure S1. Principal components analysis of gene expression in E14.5 wild-type and *Tfap2b* mutant brains.

Score plots of female embryonic SP (A) and DMH (B), male sp (C) and (D). The plots modeled above 70% of the total variance. The contributions of the first and second major components were shown along x and y axis, respectively. Female E14.5 embryos *Tfap2b*$^{+/+}$, n=3; female E14.5 embryos *Tfap2b*$^{+/-}$, n=3; male E14.5 embryos *Tfap2b*$^{+/+}$, n=3; male E14.5 embryos *Tfap2b*$^{+/-}$, n=3; SP, secondary prosencephalon; DMH, diencephalon, midbrain and hindbrain.

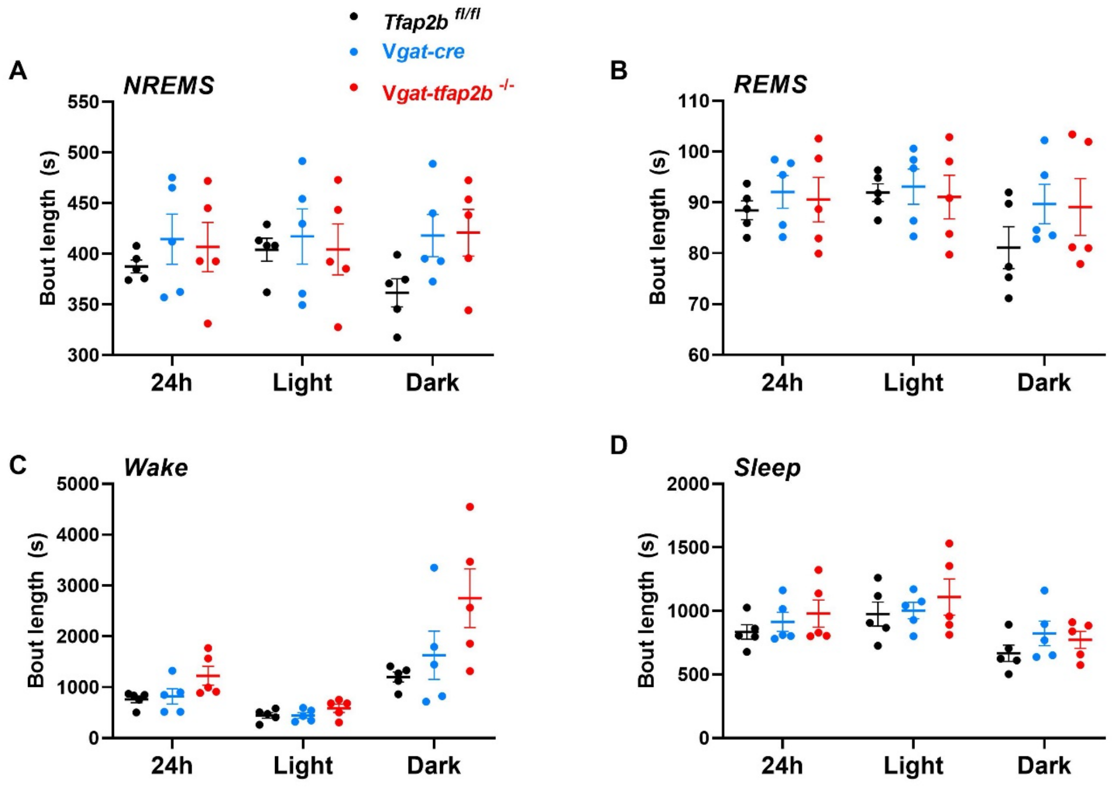

Figure S2. Sleep and wake bout lengths were not changed in female *Vgat-Tfap2b*$^{-/-}$ mice.

Bout analysis of female *Tfap2b*$^{fl/fl}$, *Vgat-cre*, *Vgat-Tfap2b*$^{-/-}$ mice during NREMS (A), REMS (B), wake (C) and sleep (D) state. Data were analyzed by two-way ANOVA followed by Sidak's multiple comparisons test and were shown as the mean ± SEM. Female *Tfap2b*$^{fl/fl}$ (n = 5), *Vgat-cre* (n = 5), *Vgat-tfap2b*$^{-/-}$ (n = 5).

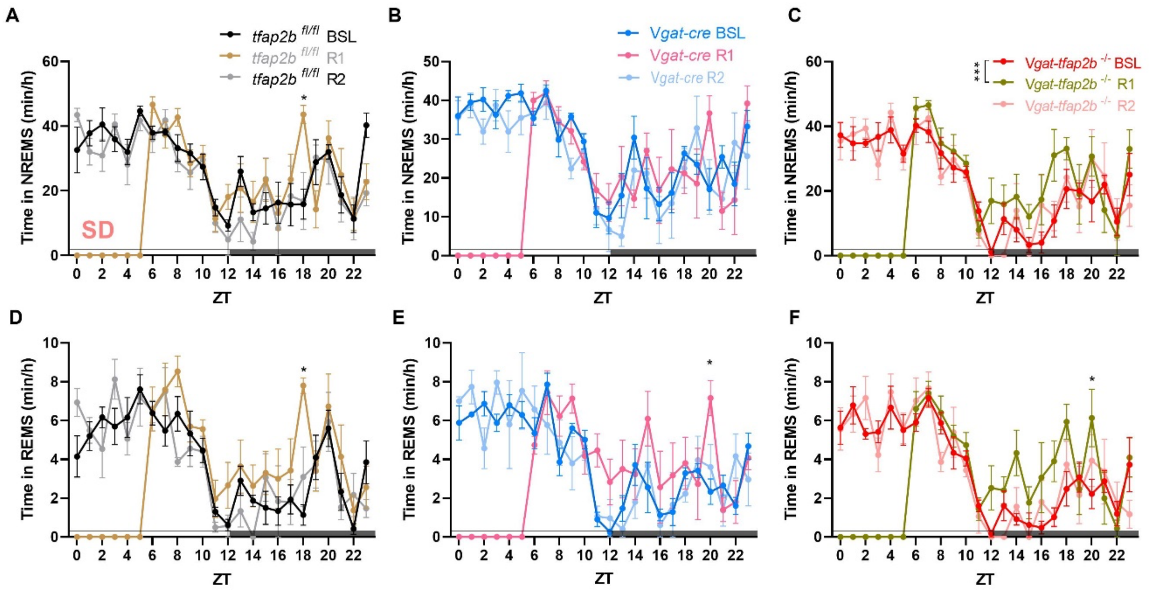

Figure S3. Sleep is increased after 6-hour sleep deprivation in all tested female mice.

NREMS quantity changes over ZT course during baseline (BSL), the first / second recovery day (R1/R2) in female *Tfap2b*$^{fl/fl}$ (A), *Vgat-cre* (B), *Vgat-tfap2b* $^{-/-}$ mice (C). REMS quantity changes over ZT course during BSL, R1, R2 in female *Tfap2b*$^{fl/fl}$ (D), *Vgat-cre* (E), *Vgat-tfap2b*$^{-/-}$ mice (F). All data were analyzed by two-way ANOVA followed by Sidak's multiple comparisons test and were shown as the mean ± SEM. Significant pairwise comparisons of BSL vs. R1 were marked with *$P < 0.05$. Female *Tfap2b*$^{fl/fl}$ (n = 5), *Vgat-cre* (n = 5), *Vgat-tfap2b*$^{-/-}$ (n = 5).

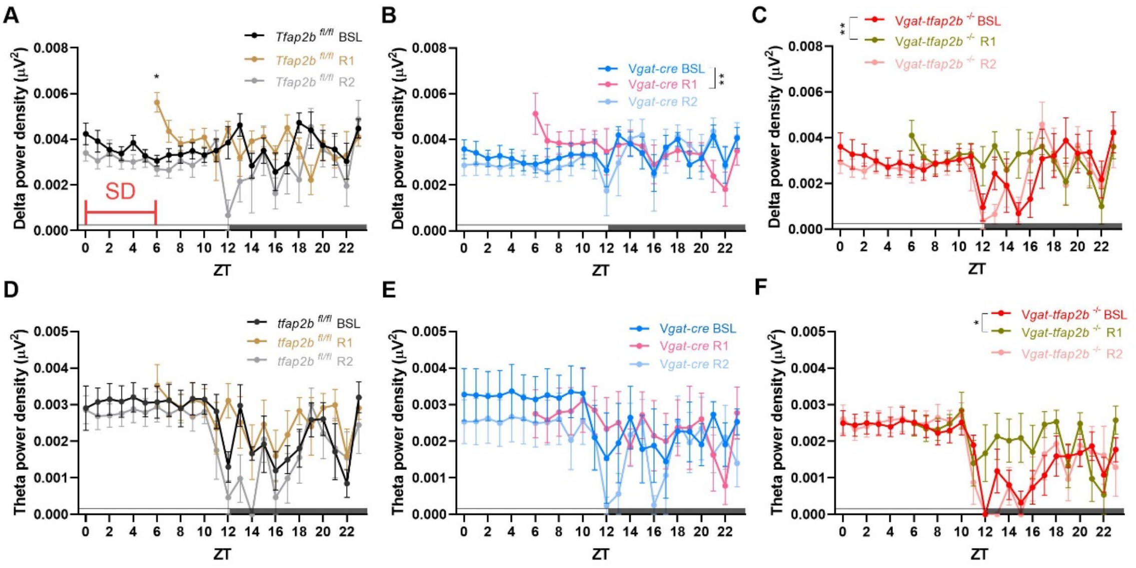

Figure S4. Weaker sleep power rebound in female *Vgat-tfap2b*$^{-/-}$ mice after 6-hour sleep deprivation.

Delta power changes over ZT course during BSL, R1, R2 in female *Tfap2b*$^{fl/fl}$ (A), *Vgat-cre* (B), *Vgat-tfap2b*$^{-/-}$ mice (C). Theta power changes over ZT course during BSL, R1, R2 in female *Tfap2b*$^{fl/fl}$ (D), *Vgat-cre* (E), *Vgat-tfap2b*$^{-/-}$ mice (F). All data were analyzed by two-way ANOVA followed by Sidak's multiple comparisons test and were shown as the mean ± SEM. Significant Time x Sleep deprivation interactive variations were specified between groups and marked with *$P < 0.05$, **$P < 0.01$; pairwise comparisons of BSL vs. R1 were marked with *$P < 0.05$. Female *Tfap2b*$^{fl/fl}$ (n = 5), *Vgat-cre* (n = 5), *Vgat-tfap2b*$^{-/-}$ (n = 5).

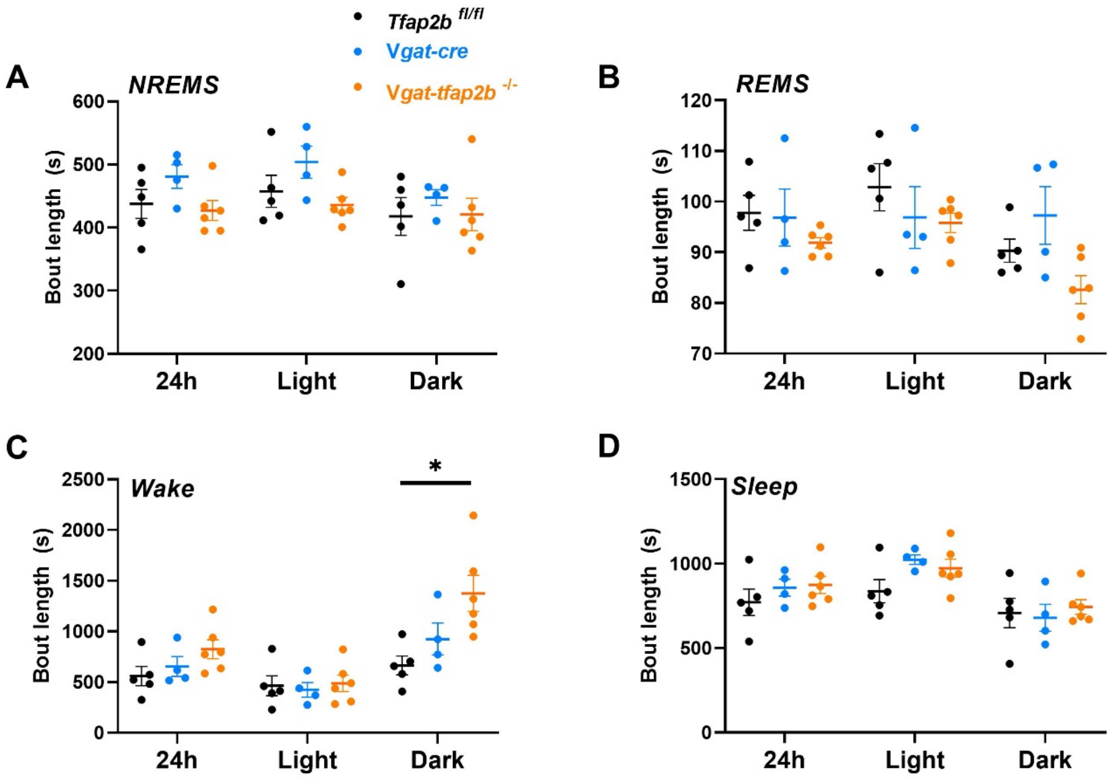

Figure S5. Sleep and wake bout lengths were not changed in the male *Vgat-fap2b*$^{-/-}$ mice.

Bout analysis of male *Tfap2b*$^{fl/fl}$, *Vgat-cre*, *Vgat-Tfap2b*$^{-/-}$ mice during NREMS (A), REMS (B), wake (C) and sleep (D) state. Data were analyzed by two-way ANOVA followed by Sidak's multiple comparisons test and were shown as the mean ± SEM. Male *Tfap2b*$^{fl/fl}$ (n = 5), *Vgat-cre* (n = 4), *Vgat-tfap2b*$^{-/-}$ (n = 6).

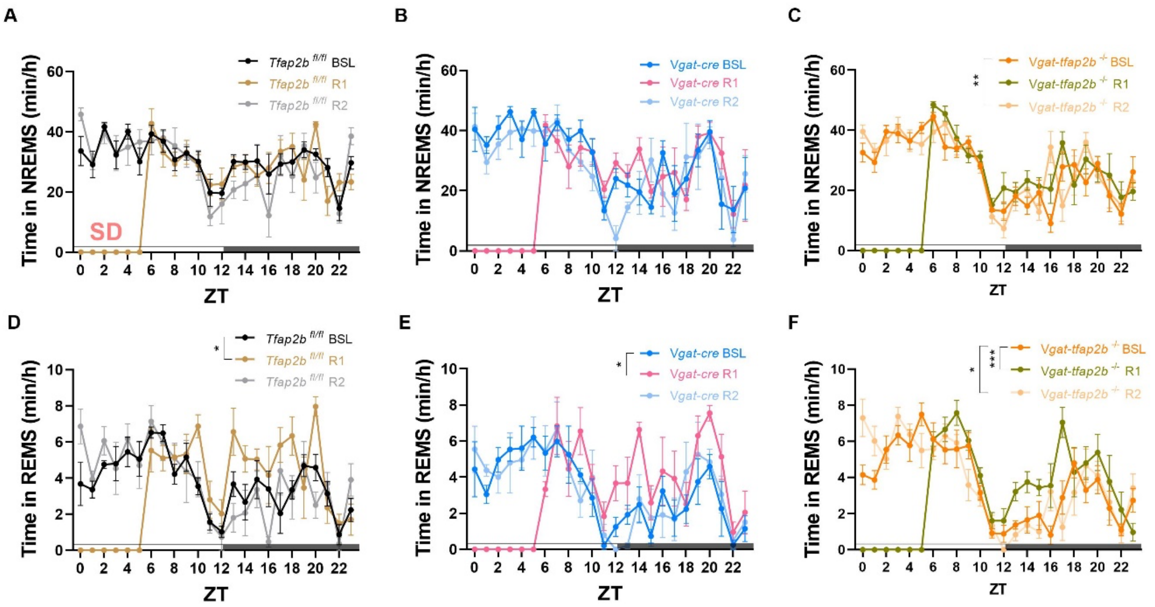

Figure S6. Sleep is increased after 6-hour sleep deprivation in the male *Vgat-tfap2b*$^{-/-}$ mutant and the *Vgat-cre* control.

NREMS quantity changes over ZT course during baseline (BSL), the first / second recovery day (R1/R2) in female *Tfap2b*$^{fl/fl}$ (A), *Vgat-cre* (B), *Vgat-tfap2b* $^{-/-}$ mice (C). REMS quantity changes over ZT course during BSL, R1, R2 in female *Tfap2b*$^{fl/fl}$ (D), *Vgat-cre* (E), *Vgat-tfap2b*$^{-/-}$ mice (F). All data were analyzed by two-way ANOVA followed by Sidak's multiple comparisons test and were shown as the mean ± SEM. Significant Time x Sleep deprivation interactive variations were specified between groups and marked with *P < 0.05, **P < 0.01, ***P < 0.001. Male *Tfap2b*$^{fl/fl}$ (n = 5), *Vgat-cre* (n = 4), *Vgat-tfap2b*$^{-/-}$ (n = 6).

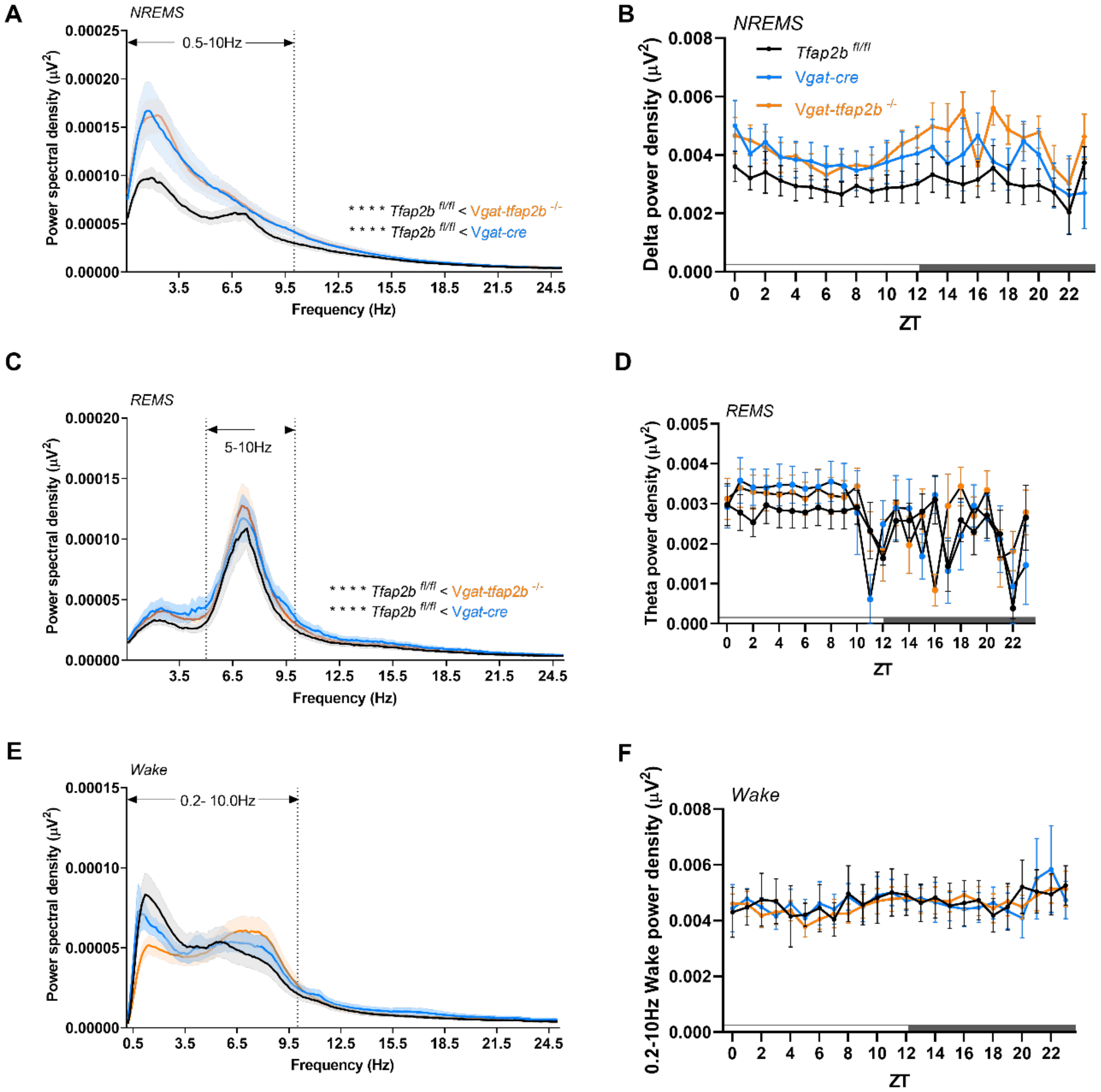

Figure S7. Sleep powers are stronger in male *Vgat-tfap2b*$^{-/-}$ mutants and the *Vgat-cre* control.

NREMS power spectra in 24h scale (A) and delta power (0.5 – 4.0 Hz) changes over ZT course (B). REMS power spectra in 24h scale (C) and theta power (5 – 10 Hz) changes over ZT course (D). Wake power spectra and 0.2- 10 Hz power changes over ZT course. Data in (A, C, E) were analyzed using Friedman test followed by Dunn's multiple comparisons test. Data in (B, D, F) were analyzed by two-way ANOVA followed by Sidak's multiple comparisons test and were shown as the mean ± SEM. Significant pairwise comparisons were marked with ****$P < 0.0001$. Male *Tfap2b*$^{fl/fl}$ (n = 5), *Vgat-cre* (n = 4), *Vgat-tfap2b*$^{-/-}$ (n = 6).

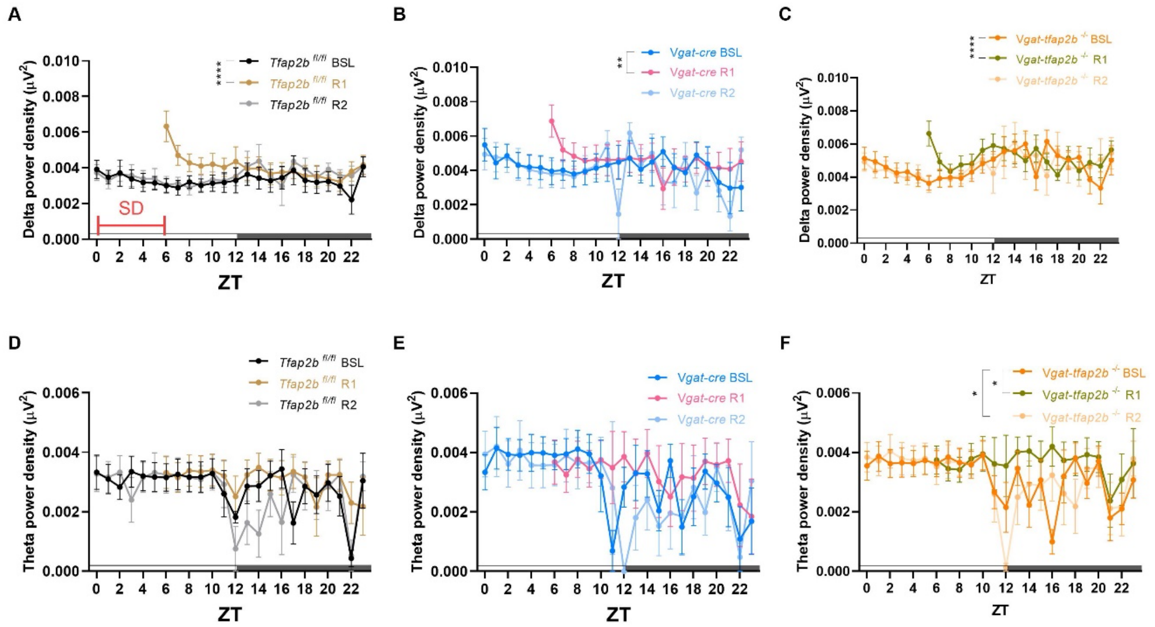

Figure S8. Sleep power rebound in both control and mutant mice after 6-hour sleep deprivation.

Delta power changes over ZT course during BSL, R1, R2 in female *Tfap2b*$^{fl/fl}$ (A), *Vgat-cre* (B), *Vgat-tfap2b*$^{-/-}$ mice (C). Theta power changes over ZT course during BSL, R1, R2 in female *Tfap2b*$^{fl/fl}$ (D), *Vgat-cre* (E), *Vgat-tfap2b*$^{-/-}$ mice (F). All data were analyzed by two-way ANOVA followed by Sidak's multiple comparisons test and were shown as the mean ± SEM. Significant Time x Sleep deprivation interactive variations were specified between groups and marked with *$P < 0.05$, **$P < 0.01$, ****$P < 0.0001$. Male *Tfap2b*$^{fl/fl}$ (n = 5), *Vgat-cre* (n = 4), *Vgat-tfap2b*$^{-/-}$ (n = 6).

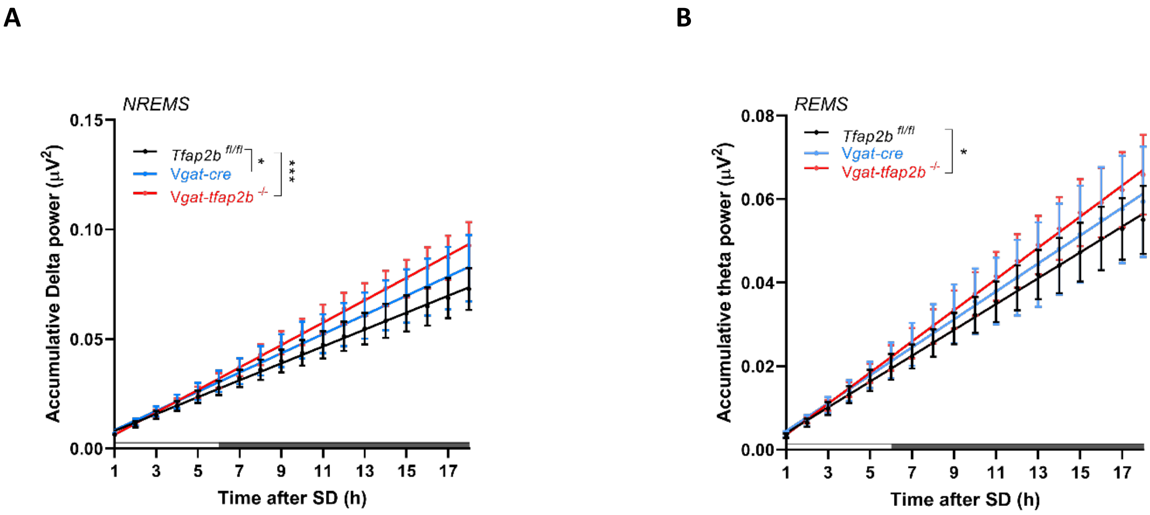

Figure S9. Sleep rebound power is stronger in the male *Vgat-tfap2b*$^{-/-}$ mutant and *Vgat-cre* control after 6-hour sleep deprivation.

Linear regression plots of accumulative delta (A) and theta power (B) after SD. Equality of slopes or intercepts was tested by simple linear regression analysis. Significantly differences were specified between groups and marked with **P* < 0.05, ****P* < 0.001, *****P* < 0.0001. Male *Tfap2b*$^{fl/fl}$ (n = 5), *Vgat-cre* (n = 4), *Vgat-tfap2b*$^{-/-}$ (n = 6).

Table S1. Genotyping primers and protocols

Gene	Primer names	Primer sequence 5' - 3'	PCR conditions	Products
[1]*Vgat-cre*	Common-F (12785)	CTTCGTCATCGGCGGCATCTG	10s at 95°, 10s at 65°(0.5°C per cycle decrease), 10s at 68° for 10 cycles;	*Vgat-cre* allele 200bp; wild-type allele 323bp
	Wildtype-R (12786)	CAGGGCGATGTGGAATAGAAA		
	Mutant-R (oIMR8292)	CCAAAAGACGGCAATATGGT	10s at 95°C, 10s at 60°C, 10s at 72°C for 28 cycles	
Tfap2b^{tm1a}	Tfap2 5'arm	GACATCCTACAATGCACAGCT	30s at 95°, 45s at 65°, 45s at 72° for 39 cycles	*Tm1a* allele 529bp (5'arm + LAR3); wild-type allele 381bp (3'arm + 5'arm)
	Tfap2 3'arm	TTGCTGTGAGCTAAGAGCTTC		
	LAR3	CAACGGGTTCTTCTGTTAGTCC		
Tfap2b^{floxed}	Tfap2 5'arm	GACATCCTACAATGCACAGCT	30s at 95°, 45s at 65°, 45s at 72° for 39 cycles	floxed allele 497bp; wild-type allele 381bp
	Tfap2 3'arm	TTGCTGTGAGCTAAGAGCTTC		
Tfap2b$^{+/-}$; *Tfap2b$^{+/+}$*	PGK-PolyA DW	CTGCTCTTTACTGAAGGCTCTTT	30s at 95°, 45s at 61°, 1min at 72° for 40 cycles	*Tfap2b-* 380bp; *Tfap2b+* 221bp
	4 Exon Rev	TTCTGAGGACGCCGCCCAGG		
	4 Exon DW	CCTCCCAAATCTGTGACTTCT		

[1]The high speed Taq DNA polymerase (KAPA2G Fast HotStart PCR-kit, KK5503) was used for the corresponding PCR condition.

Table S2. Primers and protocols used in qPCR

Gene	Forward	Reverse	qPCR conditions
eef1a1	TGCCCCAGGACACAGAGACTTCA	AATTCACCAACACCAGCAGCAA	1 min at 94°C, 30s at 62°C, 1min at 72°C for 40 cycles
Gad65	TCCGGCTTTTGGTCCTTCG	ATGCCGCCCGTGAACTTTT	
Gad67	TCCAGTGCTCTGCCATTCTG	CATAGGAGACGTCATACTGCTTGTC	
Vgat	ACCTCCGTGTCCAACAAGTC	CAAAGTCGAGATCGTCGCAGT	
